# Performance of patient-collected dried blood specimens for HIV-1 viral load testing in South Africa

**DOI:** 10.1097/QAD.0000000000004011

**Published:** 2024-09-12

**Authors:** Maitreyi Sahu, Torin Schaafsma, Adam A. Szpiro, Heidi Van Rooyen, Stephen Asiimwe, Maryam Shahmanesh, Meighan L. Krows, Nsika Sithole, Alastair Van Heerden, Ruanne V. Barnabas

**Affiliations:** a Department of Health Metrics Sciences, University of Washington, Seattle, WA; b Division of Infectious Diseases, Massachusetts General Hospital, Boston, MA; c International Clinical Research Center, Department of Global Health; d Department of Biostatistics, University of Washington, Seattle, WA, USA; e Human Sciences Research Council, Western Cape, South Africa; f Integrated Community-Based Initiatives, Kabwohe, Uganda; g Africa Health Research Institute, KwaZulu-Natal; h SAMRC/WITS Developmental Pathways for Health Research Unit, Department of Paediatrics, School of Clinical Medicine, Faculty of Health Sciences, University of the Witwatersrand, Johannesburg, Gauteng; i Center for Community Based Research, Human Sciences Research Council, Sweetwaters, KwaZulu-Natal, South Africa; j Harvard Medical School, Boston, MA, USA.

**Keywords:** dried blood spots, HIV, self-testing, validation, viral load

## Abstract

**Objective:**

Evaluate the clinical utility of patient-collected dried blood spots (DBS) in measuring HIV-1 viral load (VL) for monitoring antiretroviral therapy (ART) compared to provider-collected DBS and blood plasma.

**Design:**

In a randomized trial of community-based delivery of ART in South Africa, we assessed performance of: DBS specimens compared to plasma, and participant-collected vs. staff-collected DBS specimens, to measure HIV-1 VL.

**Methods:**

The bioMérieux NucliSENS EasyQ HIV-1 v2.0 assay was used for VL measurement. From October 2017 to November 2019, we collected 996 pairs of plasma/DBS specimens from 760 participants and 315 pairs of staff-/participant-collected DBS cards from 261 participants. We assessed DBS test sensitivity, specificity, positive predictive value (PPV) and negative predictive value (NPV) using the WHO failure threshold of 1000 copies/ml. Log-transformed VL was compared using concordance correlation coefficients (CCC) and mean differences from linear mixed models.

**Results:**

In a population with 13% detectable VL, DBS VL compared with plasma VL had 91% [95% confidence interval (CI): 86–95] sensitivity, 99% (98–100) specificity, 94% (90–98) PPV, and 99% (98–99) NPV. We observed high agreement between staff-collected DBS VL and plasma VL (CCC: 0.94), and between participant-collected DBS VL and plasma VL (CCC: 0.92). We did not observe a statistically significant difference between participant- and staff-collected DBS VL and correlation was very high (CCC: 0.97).

**Conclusions:**

VL results from participant-collected DBS are clinically comparable with those collected by clinical staff and using blood plasma. Self-collected DBS has potential for use for ART monitoring outside the clinic.

## Introduction

Monitoring of HIV-1 viral load (VL) is an essential component of managing antiretroviral therapy (ART) for individuals living with HIV/AIDS [1]. Routine VL monitoring typically consists of two tests in the first year after treatment initiation followed by annual testing for clients who are stable, allowing healthcare providers to track adherence, detect treatment failure at an early stage, monitor for drug resistance, and guide treatment adjustments [2–6]. VL testing can also inform differentiated strategies to tailor ART delivery to client needs and reduce health system burden [7–14]. However, in sub-Saharan Africa (SSA) uptake of routine VL monitoring varies widely [15–18], with reported coverage among people on ART ranging from 25% to 94% [19]. Traditionally, VL testing uses venous blood plasma, requiring phlebotomy performed by clinical staff during clinic visits, cold storage, and rapid transport to laboratories [17]. Accordingly, plasma VL testing can be expensive, particularly in low-volume rural and point-of-care settings [13,20]. Thus, there is interest in alternative VL monitoring methods that are convenient for patients and affordable for programs as a component of decentralized client-centered care [13,21,22].

One such method is the use of dried blood spots (DBS) for VL measurement, which has been well validated across studies in both high-income and resource-limited settings [22–29]. DBS specimens are collected using a finger-prick of blood, prepared by allowing blood spots to dry on a filter card, and then transported to a laboratory for nucleic acid testing [1,30]. DBS samples offer several logistical advantages, including ease of collection, stability for weeks at room temperature, and the potential to be collected by patients themselves in nonclinical settings. Whole-blood samples from DBS contain both plasma and cell-associated viral nucleic acids and can produce modestly higher levels of HIV-1 RNA than those from plasma, leading to potential overestimation which is most notable in the case of lower plasma VLs [31]. However, for detecting treatment failure, DBS performs well and is recommended by the WHO in resource-limited settings [23]. Furthermore, modeling suggests that VL-informed differentiated care using DBS is a cost-effective strategy to alleviate clinic burden in SSA [10,14]. At-home patient-collected DBS has potential to even further reduce the need for clinic visits, and recent studies from high-income countries have demonstrated feasibility of at-home self-collection of DBS for various biomarkers including HIV-1 VL [32–39]. However, in SSA the validity and clinical utility of patient-collected DBS for VL monitoring have not previously been established.

The objective of this study was to validate the use of patient-collected DBS specimens for HIV-1 VL monitoring which were collected as part of a field-based randomized trial of community delivery of ART in South Africa [40]. To fulfill this aim, we compared VL measurements from participant-collected DBS, staff-collected DBS, and blood plasma collected in the community setting. This study evaluates whether patient-collected DBS can serve as a clinically useful tool for VL monitoring as part of decentralized HIV programs in SSA.

## Methods

The DBS VL validation was a preplanned sub-study embedded in a randomized trial of community-based delivery of ART in KwaZulu-Natal, South Africa (the DO ART Study) [40].

Participants living with HIV enrolled in the community-based ART arm of the DO ART Study provided venous blood for plasma VL and up to two DBS specimens (one staff-collected, and one self-collected) during their ART monitoring visits. Pairs of DBS specimens were collected as follows: study staff collected an initial DBS card while training participants on safe collection of DBS including by providing written information with pictures, and participants then independently used a lancet to self-collect a second DBS card with supervision at the same visit. Cards were kept at room temperature that day then brought to the study site for packaging.

### Laboratory Methods

Specimens were picked up daily by the laboratory courier service and transported 100–250 km to Global Labs (Durban), where VL was measured using the bioMérieux NucliSENS EasyQ HIV-1 v2.0 assay, which has a plasma VL lower limit of quantification of 20 copies/ml and a DBS VL lower limit of quantification of 100 copies/ml.

### Analysis

Across specimen pairs, we assessed performance of: DBS specimens compared to blood plasma, and self-collected vs. staff-collected DBS specimens. We assessed test sensitivity, specificity, positive predictive value (PPV) and negative predictive value (NPV) for detectable VL at the WHO threshold of 1000 copies/ml, assuming a 13% prevalence of virologic failure [41]. Agreement between log_10_-transformed VL measurements was examined using the concordance correlation coefficient for repeated measurements (CCC), which is designed to quantify correlation and detect deviations from the one-to-one line [42]. All values below the DBS VL lower limit of quantification (100 copies/ml) were assigned half that value (50 copies/ml). In addition, in a subset of specimens where either result was detectable, we used Bland–Altman methods [43,44] and assessed mean differences and limits of agreement using linear mixed models accounting for repeated measures. Analyses were done in R using the SimplyAgree package [45].

### Ethics

All participants provided written informed consent prior to enrollment. The DO ART Study was approved on 2/16/2016 by the Human Sciences Research Council Research Ethics Committee in South Africa and the University of Washington Institutional Review Board in Seattle, USA (REC6/13/11/15).

## Results

From October 2017 to November 2019, specimens were collected from 767 participants at 1007 visits (S1 Table 1, Supplemental Digital Content). Pairs of plasma and staff-collected DBS results were available from 760 participants at 996 visits; DBS self-collection began in November 2018 and pairs of self- and staff-collected DBS results were available from 261 participants at 315 visits. Table 1 provides baseline participant characteristics for the DBS and participant-collected DBS validation studies.

**Table 1:** Baseline characteristics of study participants providing dried blood spots (DBS) for HIV-1 viral load (VL) monitoring in the DO ART Study in South Africa.

		DBS vs. plasma VL validation		Participant vs. staff-collected DBS VL validation	
		*N* = 760	%	*N* = 261	%
Sex	Women	430	(57%)	134	(51%)
	Men	330	(43%)	127	(49%)
Age (years)	18–29	256	(34%)	102	(39%)
	30–49	448	(59%)	145	(56%)
	≥ 50	56	(7%)	14	(5%)
CD4^+^ ell count (cells/μl, baseline)	100–349	234/754	(31%)	106/260	(41%)
	350–499	158/754	(21%)	64/260	(25%)
	500+	362/754	(48%)	90/260	(35%)

Notes: For the DBS/plasma validation, 996 paired samples were evaluated using staff-collected DBS and plasma VL from 760 participants. For the participant/staff DBS validation, 315 paired samples were evaluated using patient- and staff-collected DBS VL from 261 participants. A total of 254 participants contributed to both validations.

**Fig. 1:**
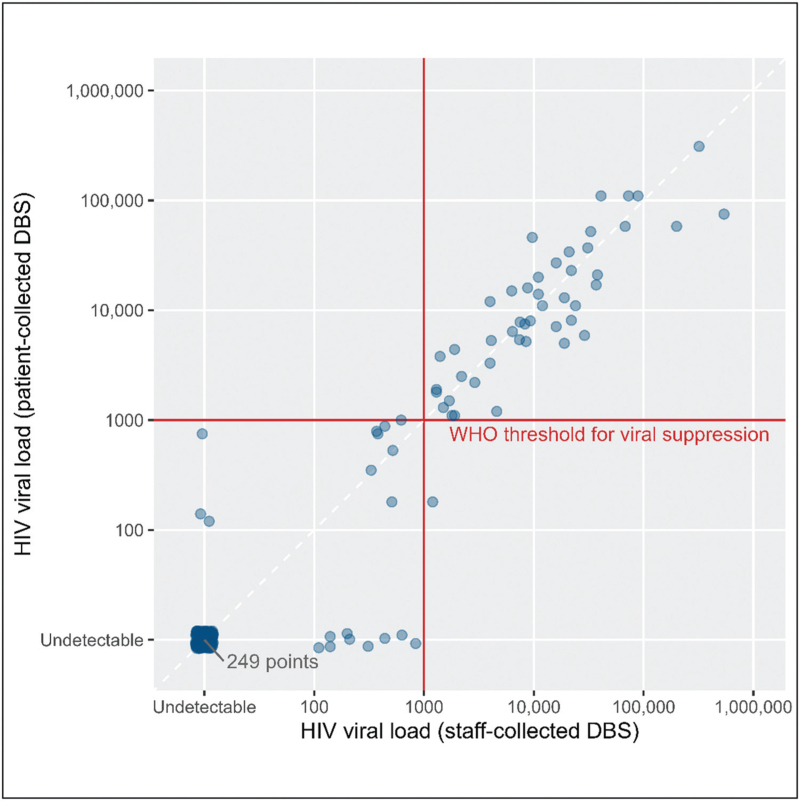
HIV-1 viral load measurements for participant-collected dried blood spots (DBS) compared with staff-collected DBS in the DO ART Study in South Africa (copies/ml).

Notes: HIV-1 viral load measurements are from 315 pairs of staff-/participant-collected DBS cards from 261 participants.

First, we compared DBS VL with plasma VL (S2 Figure 1, Supplemental Digital Content). Among 996 plasma/DBS pairs, agreement was high (CCC: 0.95). Using mixed-effects linear regression, we estimated that on average DBS and plasma VL measurements differed by −0.36 log_l0_ copies/ml [95% confidence interval (CI): −0.43 to −0.29] among 272 pairs where either result was detectable (S2 Figure 2, Supplemental Digital Content). Using the WHO treatment failure threshold, DBS VL compared with plasma VL had 91% (86–95) sensitivity, 99% (98–100) specificity, 94% (90–98) PPV, and 99% (98 –99) NPV in a population with 13% detectable VL. Appendix S3 shows the two-by-two tables using the WHO threshold (S3 Table 1, Supplemental Digital Content) and the DBS limit of quantification (S3 Table 2, Supplemental Digital Content).

Next, we compared staff- and patient-collected DBS VL with plasma VL for 254 participants. Using the WHO failure threshold, we observed high concordance between staff-collected DBS VL and plasma VL (CCC: 0.94), and between participant-collected DBS VL and plasma VL (CCC: 0.92).

Finally, we compared participant-collected DBS VL with staff-collected DBS VL (Fig. 1). Across 315 pairs of patient-/staff-collected DBS cards, concordance was very high (CCC: 0.97). Among 66 specimen pairs where either result was detectable, we did not observe a significant difference between staff- and participant-collected DBS VL using a linear mixed model (mean difference −0.10 log_l0_ copies/ml [-0.21 to 0.02]) (S2 Figure 3, Supplemental Digital Content). At the WHO failure threshold only two pairs (0.6%) were discrepant (S3 Table 3, Supplemental Digital Content); at the limit of quantification, 12 pairs (4%) were discrepant (S3 Table 4, Supplemental Digital Content). We did not observe meaningful differences by sex or CD4^+^ cell count.

## Discussion

The WHO recommends clinic staff-administered DBS for VL monitoring and detection of treatment failure [1]. Our study shows that patient-collected DBS produces comparable results. Specifically, our results show that patient-collected DBS HIV-1 VL has high agreement with DBS collected by clinic staff (CCC: 0.97), and both clinic- and patient-collected DBS have high agreement with plasma VL (CCC: 0.94 and 0.92, respectively). Furthermore, DBS VL compared with plasma VL performs well (with >90% sensitivity, specificity, PPV, and NPV), in line with existing evidence supporting the WHO recommendation to use DBS to detect treatment failure in low-resource settings [22–28]. Altogether, these results indicate that patient-collected DBS can be leveraged for accurate, rapid, and decentralized monitoring in the southern African setting.

To our knowledge, this is the first study assessing performance of patient-collected DBS for HIV-1 VL monitoring in a low- or middle-income setting. While our results validate the clinical potential of patient-collected DBS, the extension to at-home self-collection is not yet clear. Studies from high-income countries suggest that DBS self-collection holds promise but may pose challenges [32–39]. In Canada, at-home self-collected DBS was successfully used for population-based SARS-CoV-2 antibody testing [46,47]. However, in the US pilot feasibility studies of at-home self-collected DBS for HIV-1 VL monitoring among men who have sex with men had mixed results: one study concluded DBS was feasible and had potential for longitudinal VL monitoring, but another found that 27% of DBS cards were rated as bad quality [37–39]. Authors noted the importance of providing clear written and video instructions including close-up images of the puncture site [39]. Home-based self-collected DBS should be further evaluated in a resource-limited context, including evaluating the feasibility, test performance, and longitudinal impact on costs and outcomes for routine VL monitoring and VL-informed differentiated care, and as part of a package of integrated self-care services provided outside the clinic.

Though DBS for HIV-1 VL has been well validated in both high- and low-resource settings [22–28], there are some potential drawbacks compared to plasma VL. A meta-analysis found that across 40 studies evaluating commonly used VL assays, DBS had a sensitivity of 93% and specificity of 87% compared with plasma VL, with poorer specificity at lower levels of plasma VL [23]. Our study finds a sensitivity of 91% but higher specificity of 99%. While this generally indicates good test performance, we should be aware that the sensitivity indicates that 7–9% of people failing treatment will be missed by DBS and may only be detected a year later during follow-up VL testing, allowing time for viral resistance to develop. Furthermore, the reduced specificity corresponding to over-quantification of virologic failure due to cellular material with HIV RNA/DNA in whole blood specimens could increase unnecessary follow-up care. Regardless, DBS is recommended by the WHO in settings with limited infrastructure for plasma VL given evidence that VL monitoring using DBS can cost-effectively improve outcomes while minimizing burdens on health systems and patients [6,7,10,13].

This study had both strengths and limitations. Key strengths of this study were its prospective design and novelty in its potential implication for at-home VL monitoring outside high-income settings. Beyond known limitations of DBS, the key limitation of this study is that clinical staff were present during patient collection of DBS specimens and provided one-on-one training, so we cannot extrapolate feasibility and validity to at-home unsupervised collection of DBS samples. However, we find that with limited training the participants were well equipped to collect their own DBS specimens. Finally, this study evaluates only test performance but not the downstream impact following a first result showing detectable VL including time to follow-up VL or treatment switches following confirmed virologic failure – future studies of self-collected DBS could longitudinally consider the VL cascade of care [18].

Across HIV programs in SSA, scale-up of routine VL monitoring has been slow due to logistical challenges and high per-test costs in low-volume settings [17,20]. Participant-collected DBS, if effective when collected at home, has potential to be a high-value component of HIV programs by reducing clinic visit load, requiring less burden on clients and health systems [9,10].

## Conclusions

We find that participant-collected DBS performs well in a community setting, opening the door for research on home-based self-collected DBS, which could further streamline services as part of a flexible, decentralized approach to VL monitoring for ART adherence and response in SSA. At-home self-collected DBS should be evaluated in resource-limited settings for ART monitoring and as part of VL-informed differentiated care.

## Acknowledgements

We thank the DO ART Study participants and the members of the Data and Safety Monitoring Board. We also thank Peter Ehrenkranz for his valuable input on the design of the study. This work was completed with support from the Bill and Melinda Gates Foundation (BMGF #OPP1134599).

Funding: This work was completed with support from the Bill and Melinda Gates Foundation.

Author contributions: R.V.B., H.v.R., and A.v.H. conceptualized and designed the study. S.A., M.W., M.L.K., N.S., and A.v.H. conducted the study. T.S. and A.A.S. analyzed the data. M.S. wrote the manuscript draft. All authors critically reviewed and approved the manuscript.

### Conflicts of interest

There are no conflicts of interest.

## Supplementary Material

Supplemental Digital Content
