# Supplementary material for: Performance of patient-collected dried blood specimens for HIV-1 viral load testing in South Africa
Source: AIDS. 2024 Sep 12;38(15):2050–5. doi: 10.1097/QAD.0000000000004011 (PMC11562487; doi:10.1097/QAD.0000000000004011)
Supplement: Supplemental Digital Content [file aids-38-2050-s001.docx]

**DO ART DBS VALIDATION STUDY**

**SUPPLEMENTARY MATERIAL**

**Appendix S1: Study enrollment**

- S1, Table 1: Overview of study participants and collected samples for DBS validation

**Appendix S2: Additional Figures**

- S2, Figure 1: HIV-1 viral load measurements for DBS compared with plasma (copies/mL)
- S2, Figure 2: Bland-Altman plot of agreement between HIV-1 VL for DBS compared with plasma, for pairs with at least one detectable value
- S2, Figure 3: Bland-Altman plot of agreement between HIV-1 VL using participant-collected versus staff-collected DBS, for pairs with at least one detectable value

**Appendix S3: Two-by-two contingency tables**

- S3, Table 1: Two-by-two table comparing HIV-1 VL for DBS compared with plasma using the WHO failure threshold (1000 copies/mL)
- S3, Table 2: Two-by-two table comparing HIV-1 VL for DBS compared with plasma using the DBS limit of quantification (100 copies/mL)
- S3, Table 3: Two-by-two table comparing HIV-1 VL for participant-collected DBS compared with staff-collected DBS using the WHO failure threshold (1000 copies/mL)
- S3, Table 4: Two-by-two table comparing HIV-1 VL for participant-collected DBS compared with staff-collected DBS using the DBS limit of quantification (100 copies/mL)

**S1, Table 1: Overview of study participants and collected samples for DBS validation in the DO ART Study in South Africa**

| **Validation study** | DBS/plasma validation only | Both validations | Self-/staff-collected DBS validation only | Total |
| --- | --- | --- | --- | --- |
| **Results available** | Plasma & staff-collected DBS only (2) | Plasma, staff-collected DBS, & participant-collected DBS (3) | Self- & staff-collected DBS (2) | -- |
| **Visit count** | 692 visits | 304 visits | 11 visits | 1007 visits |
| **Participant count** | 506 distinct participants | 254 distinct participants | 7 distinct participants | 767 distinct participants |

**S2, Figure 1: HIV-1 viral load measurements for dried blood spots (DBS) compared with plasma in the DO ART Study in South Africa (copies/mL)**


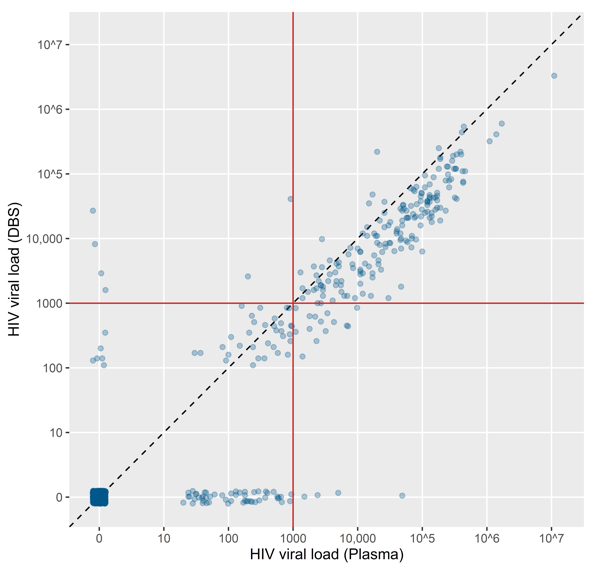


*Notes: HIV-1 viral load measurements are from 996 pairs of staff-/participant-collected DBS cards from 760 participants.*

**S2, Figure 2: Bland-Altman plot of agreement between HIV-1 VL measurements for DBS compared with plasma, for pairs with at least one detectable value, in the DO ART Study in South Africa (N=272)**


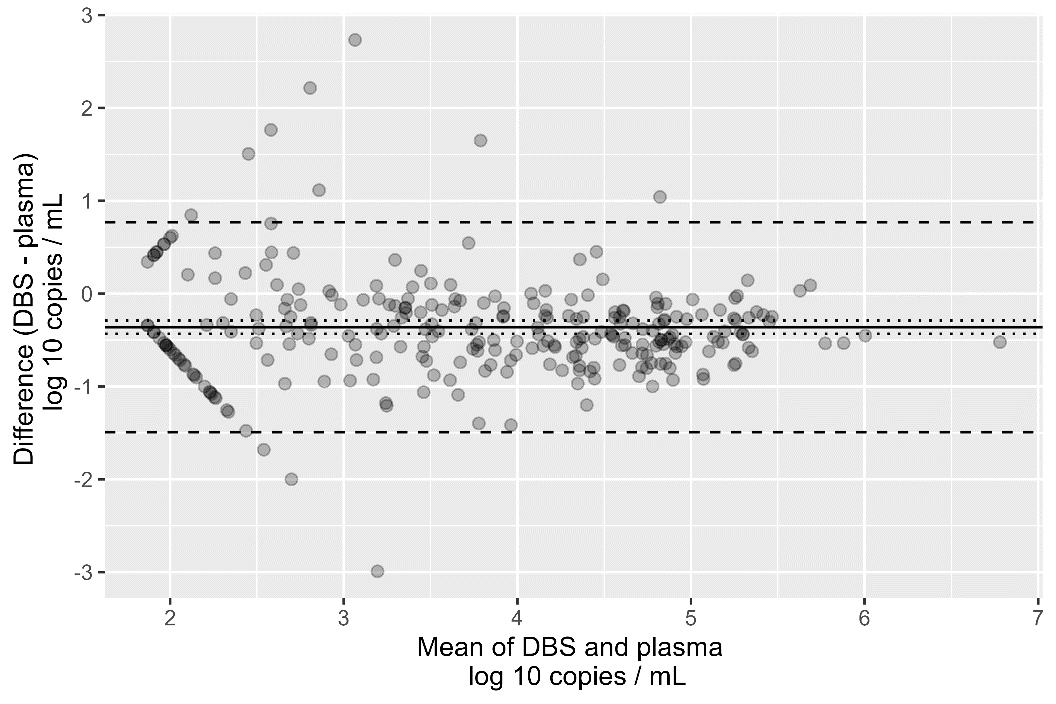


*Notes: The solid line represents the mean difference, the dotted lines represent the 95% confidence interval and the dashed lines represent the Bland-Altman limits of agreement.*

**S2, Figure 3: Bland-Altman plot of agreement between HIV-1 VL measurements using participant-collected versus staff-collected DBS, for pairs with at least one detectable value, in the DO ART Study in South Africa (N=66)**


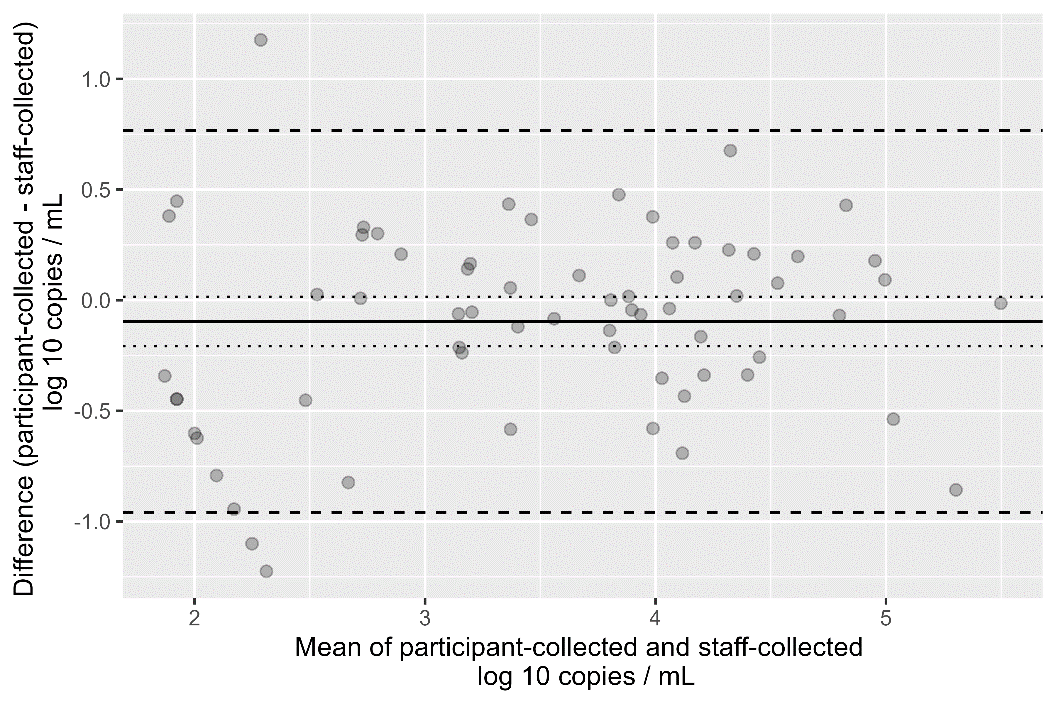


*Notes: The solid line represents the mean difference, the dotted lines represent the 95% confidence interval and the dashed lines represent the Bland-Altman limits of agreement.*

**S3, Table 1: Two-by-two table comparing HIV-1 VL for staff-collected DBS compared with plasma using the WHO failure threshold (1000 copies/mL)**

|  | Number of paired samples,  with VL measured using plasma | | |
| --- | --- | --- | --- |
| VL measured using DBS | Plasma  < 1000 | Plasma  ≥ 1000 | All |
| DBS < 1000 | 791 | 18 | 809 |
| DBS ≥ 1000 | 6 | 181 | 187 |
| All | 797 | 199 | 996 |

**S3, Table 2: Two-by-two table comparing HIV-1 VL for staff-collected DBS compared with plasma using the DBS limit of quantification (100 copies/mL)**

|  | Number of paired samples,  with VL measured using plasma | | |
| --- | --- | --- | --- |
| VL measured using DBS | Plasma  < 100 | Plasma  ≥ 100 | All |
| DBS < 100 | 724 | 32 | 756 |
| DBS ≥ 100 | 14 | 226 | 240 |
| All | 738 | 258 | 996 |

**S3, Table 3: Two-by-two table comparing HIV-1 VL for participant-collected DBS compared with staff-collected DBS using the WHO failure threshold (1000 copies/mL)**

|  | Number of paired samples,  with VL measured staff-collected DBS | | |
| --- | --- | --- | --- |
| VL measured using participant-collected DBS | Staff-collected DBS <1000 | Staff-collected  DBS ≥ 1000 | All |
| Patient-collected DBS < 1000 | 267 | 1 | 268 |
| Patient-collected DBS ≥ 1000 | 1 | 46 | 47 |
| All | 268 | 47 | 315 |

**S3, Table 4: Two-by-two table comparing HIV-1 VL for participant-collected DBS compared with staff-collected DBS using the DBS limit of quantification (100 copies/mL)**

|  | Number of paired samples,  with VL measured staff-collected DBS | | |
| --- | --- | --- | --- |
| VL measured using participant-collected DBS | Staff-collected DBS <100 | Staff-collected  DBS ≥ 100 | All |
| Patient-collected DBS < 100 | 249 | 9 | 258 |
| Patient-collected DBS ≥ 100 | 3 | 54 | 57 |
| All | 252 | 63 | 315 |
